# A non-parametric approach for detecting gene-gene interactions associated with age-at-onset outcomes

**DOI:** 10.1186/1471-2156-15-79

**Published:** 2014-07-01

**Authors:** Ming Li, Joseph C Gardiner, Naomi Breslau, James C Anthony, Qing Lu

**Affiliations:** 1 Division of Biostatistics, Department of Pediatrics, University of Arkansas for Medical Sciences, Little Rock, AR 72202, USA; 2 Department of Epidemiology and Biostatistics, Michigan State University, East Lansing, MI 48824, USA

**Keywords:** Weighted Nelson-Aalen, Cox regression, Progression of nicotine dependence, Joint association

## Abstract

**Background:**

Cox-regression-based methods have been commonly used for the analyses of survival outcomes, such as age-at-disease-onset. These methods generally assume the hazard functions are proportional among various risk groups. However, such an assumption may not be valid in genetic association studies, especially when complex interactions are involved. In addition, genetic association studies commonly adopt case-control designs. Direct use of Cox regression to case-control data may yield biased estimators and incorrect statistical inference.

**Results:**

We propose a non-parametric approach, the weighted Nelson-Aalen (WNA) approach, for detecting genetic variants that are associated with age-dependent outcomes. The proposed approach can be directly applied to prospective cohort studies, and can be easily extended for population-based case-control studies. Moreover, it does not rely on any assumptions of the disease inheritance models, and is able to capture high-order gene-gene interactions. Through simulations, we show the proposed approach outperforms Cox-regression-based methods in various scenarios. We also conduct an empirical study of progression of nicotine dependence by applying the WNA approach to three independent datasets from the Study of Addiction: Genetics and Environment. In the initial dataset, two SNPs, rs6570989 and rs2930357, located in genes *GRIK*2 and *CSMD*1*,* are found to be significantly associated with the progression of nicotine dependence (ND). The joint association is further replicated in two independent datasets. Further analysis suggests that these two genes may interact and be associated with the progression of ND.

**Conclusions:**

As demonstrated by the simulation studies and real data analysis, the proposed approach provides an efficient tool for detecting genetic interactions associated with age-at-onset outcomes.

## Background

For most common complex diseases, if not all, the currently identified genetic loci only explain a small percentage of the disease heritability [1,2]. The search for genetic variants underlying complex diseases remains to be a major goal and challenge for the coming decades. While genetic variants, such as rare variants and structure variation, may contribute to the remaining heritability, part of the missing heritability could be explained by the interplay of genetic variants through complicated mechanisms [3,4]. The study of gene-gene interaction may reveal novel findings explaining missing heritability, and shed light on the underlying etiological and pathophysiological processes that result in complex diseases. Although substantial efforts are given to identification of gene-gene (G-G) interactions related to binary disease outcomes, less attention has been given to G-G interaction research on other clinical features, such as ages at disease onset, which may more closely reflect the dynamic process of disease development [5]. A number of studies show that the intensity of natural selection on a gene declines with the age at which it is expressed [6-8]. In addition, practical evidence suggests that many complex diseases, such as Alzheimer’s diseases and bipolar diseases, have separate pools of genetic variants that contribute to the early-onset and late-onset cases, indicating distinctive biological pathways involved in the disease development process [9,10]. Though G-G interactions are ubiquitous in various biological pathways related with the disease development process [3], identification of these G-G interactions presents continuing challenges.

Cox-regression is a powerful tool for the genetic analysis of age-at-disease-onset outcomes. It has been used in genetic association studies for detecting single genetic variant associated with disease progression [11-13]. To better characterize the association in a candidate gene/region, Cox-regression has also been extended to handle multiple loci via haplotype analysis [14-16]. However, Cox-regression is less suitable for the analysis of a large number of genetic variants and possible G-G interactions, because of the rapidly increasing number of parameters. In addition, Cox regression assumes the hazard rates are proportional among various risk groups. Such an assumption may be questionable in genetic studies, especially when complex interactions are involved. Further, most of the current genetic association studies adopt case-control designs, by which cases are usually over-sampled from the source population. Applying Cox regression to case-control studies may raise another issue of biased estimation of hazard ratios [17,18]. To address this issue, Nan *et al.* proposed to analyze age-at-disease-onset for case-control studies using a modified case-cohort approach (MCC) [19]. It was shown that this approach had very small bias when the disease prevalence was low. However, the bias would increase with the disease prevalence, which may limit its application to studies of common diseases.

As a non-parametric alternative to Cox regression, Nelson-Aalen estimator has been widely used to analyze survival outcomes. It was first introduced by Nelson and was later on rediscovered by Aalen, who derived the estimator using the modern counting process techniques [20,21]. It was shown to have a number of nice properties, such as requiring less assumptions and better small-sample-size performance than other standard approaches [22]. Considering these advantages, we propose a weighted Nelson-Aalen (WNA) approach for detecting genetic variants associated with age-at-disease-onset, considering possible interactions. The proposed approach searches for the best combination of loci forwardly, and tests their joint association with the age-at-disease-onset. Our approach has the following advantages. First, it is a multi-locus approach that is applicable to a set of genetic variants (i.e. from a number of candidate genes or a genetic pathway) with the consideration of high-order interaction. Second, it is a non-parametric approach, which does not make any assumption of the disease models. Third, it can be directly applied to prospective cohort studies, and can be easily extended for population-based case-control studies. Through simulations, we compare the performance of the proposed approach with Cox-regression-based approaches under both perspective cohort and case-control study designs. We further illustrate our approach with a real data application to smokers’ progression to nicotine dependence (ND), using three independent datasets from the Study of Addiction: Genetics and Environment (SAGE).

## Methods

Suppose we have a study population of *n* subjects, which is a sub-cohort from the source population. We denote T_*i*_, δ_*i*_ and *X*_*i*_ as the observed age, the disease status and the genetic markers for subject *i*, respectively. Let $T_{i}=\min\left(T_{i}^{O},T_{i}^{S}\right)$, where $T_{i}^{O}$ and $T_{i}^{S}$ are the age-at-disease-onset and age-at-survey for subject *i*. Then $T_{i}^{O}$ is either observed or right-censored, and we assume the cause of censoring is independent with either age-at-disease-onset or any genetic variants. Further denote $\delta_{i}=I\left(T_{i}^{O}\leq T_{i}^{S}\right)$, where *I*(.) is an indicator function. Without loss of generality, we assume *K* genetic markers, *X*_i_ = (*X*_*i*1_, *X*_*i*2_......, *X*_*iK*_), are single nucleotide polymorphisms (SNP) with three possible genotypes, *X*_*ij*_ ∈ {AA, Aa, aa}, 1 ≤ *j* ≤ *K*. Our hypothesis is that the *K* SNPs and their possible interactions may be associated with the age-at-disease-onset outcome. In the following, we introduce the WNA approach for perspective cohort studies, and then extend it for population-based case-control studies.

### Weighted nelson-Aalen for prospective cohort studies

#### a. Association testing of multiple genotype groups with age-at-disease-onset

Assume *k* disease-susceptibility SNPs are associated with ages of disease onset, by forming *L* G-G groups, *G*_1_, *G*_2_,......,*G*_*L*_, each representing a different hazard rate for the disease onset. Given these G-G groups, we can partition all subjects into *L* groups, *S*_1_, *S*_2_ ......,*S*_*L*_, where *S*_1_ = {*i*|*X*_*i*_ = *G*_*l*_}, 1 ≤ *l* ≤ *L*. Suppose the onsets of a disease are observed at *M* distinct ages, *t*_1_ < *t*_2_ < ...... < *t*_*M*_. Let *Y* (*t*) be the number of subjects who are at risk at age *t*, and *N*(*t*) be the number of subjects who have disease-onset by age *t*. The cumulative hazard function *H* and survival function *S* are estimated as,

(1) $$\begin{array}{ll} \hat{H}\left(t\right)=\int_{0}^{t}\{Y\left(u\right)\}{}^{-1}\mathrm{dN}\left(u\right) & =\sum_{u\leq t}\frac{\Delta N\left(u\right)}{Y\left(u\right)},\\ \hat{S}\left(t\right) & =\exp\left(-\hat{H}\left(t\right)\right) \end{array}$$

In a similar manner, we can also define the group-specific cumulative hazard functions *H*_*l*_ (*t*), 1 ≤ *l* ≤ *L*, based on the subjects within group *S*_*l*_. To examine the joint association of *k* SNPs with the age-at-disease-onset, we test the following hypothesis: *H*_0_ : *H*_1_(*t*) = *H*_2_(*t*)=...... = *H*_*L*_(*t*) for all *t* ≤ *τ* versus *H*_*A*_ : at least one *H*_*l*_(*t*) is different for some *t* ≤ *τ*.

Here *τ* is the largest observed age of onset in the study.

Let *Y*_*l*_ (t) be the number of subjects in group *S*_*l*_ who are at risk at age *t*; *N*_*l*_ (*t*) be the number of subjects in group *S*_*l*_ who have the disease-onset by age *t*. The test statistic can be formed based on *Z* = (*Z*_1_, *Z*_2_,...... *Z*_*L*_), where

(2) $$Z_{l}=\int_{0}^{\tau }W\left(t\right)\left(dN_{l}\left(t\right)-\frac{Y_{l}\left(t\right)}{Y\left(t\right)}\mathrm{dN}\left(t\right)\right),1\leq l\leq L;$$

In Equation (2), *W* (*t*) is a weight function for the ages at disease onset. A variety of weight functions have been proposed in the literature. For example, *W* (*t*) = 1 for any *t*, would lead to the widely used log-rank test [23]; *W* (*t*) = *Y*(*t*) would lead to Mann-Whitney-Wilcoxon test and the generalization of Kruskal-Wallis test [24,25]. In our study, we suggest using the weight function,

(3) $$W\left(t\right)=\prod_{s\leq t}\left(1-\frac{\Delta N\left(s\right)}{Y\left(s\right)+1}\right),$$

which has a form similar to the Kaplan-Meier estimator in the entire study population, and gives the most weight to early disease onset. This weight function was first proposed by Peto *et al.*[26], and was also suggested in a series of articles [27,28].

The variance of *Z*_*l*_ and the covariance between *Z*_*l*_ and *Z*_*l*_′ can then be calculated as,

(4) $$\begin{array}{ll} \mathrm{Var}\left(Z_{l}\right)= & \int_{o}^{\tau }W{\left(t\right)}^{2}\frac{Y_{l}\left(t\right)}{Y\left(t\right)}\left(1-\frac{Y_{l}\left(t\right)}{Y\left(t\right)}\right)\\ & \times \left(\frac{Y\left(t\right)-\Delta N\left(t\right)}{Y\left(t\right)-1}\right)\mathrm{dN}\left(t\right),1\leq l\leq L; \end{array}$$

(5) $$\begin{array}{ll} \mathrm{Cov}\left(Z_{l},Z_{l^{\prime }}\right)= & -\int_{o}^{\tau }W{\left(t\right)}^{2}\frac{Y_{l}\left(t\right)}{Y\left(t\right)}\\ & \times \frac{Y_{l^{\prime }}\left(t\right)}{Y\left(t\right)}\left(\frac{Y\left(t\right)-\Delta N\left(t\right)}{Y\left(t\right)-1}\right)\mathrm{dN}\left(t\right),1\leq l\neq l^{\prime }\leq L. \end{array}$$

The components of Z = (*Z*_1_, *Z*_2_,......, *Z*_*L*_) are linearly dependent because $\sum_{l=1}^{L}Z_{l}=0$. Therefore, the test statistics can be calculated based on any *L -* 1 components of *Z*, such as *Z*_1_, *Z*_2_,......, *Z*_*L-*1_. The test statistic can be formed as,

(6) $$\Delta_{\mathrm{WNA}}=\left(Z_{1},Z_{2},......,Z_{L-1}\right)\sum^{-1}{\left(Z_{1},Z_{2},......,Z_{L-1}\right)}^{\prime },$$

where Σ is the variance-covariance matrix for (*Z*_1_, *Z*_2_,......, *Z*_*L-*1_). Under the null hypothesis of no association, the above test statistic has asymptotically a Chi-square distribution with *L -* 1 degrees of freedom. The theoretical details of the test can be found elsewhere [29].

#### b. Selection of multi-SNP combinations by recursive partitioning

In genetic association studies with a set of SNPs, it is expected that only a small subset of SNPs are associated with the disease. To determine the disease-susceptibility SNPs and the associated G-G groups, we adopt a recursive partitioning algorithm. The algorithm starts with a null model by treating all study samples as one group. In each of the following steps, it gradually selects disease-susceptibility markers, and then divides samples into different G-G groups. In step one, we search among all available SNPs for a single SNP that can best divide samples into two groups. Assuming the minor allele a is the risk allele of *j-*th SNP, we consider three partitioning strategies:

1) Dominant effect:

$$\left\{S_{1}^{j}=\left\{i|X_{i,j}=\mathrm{AA}\right\},S_{2}^{j}=\left\{i|X_{i,j}=\mathrm{Aa}\;\mathrm{or}\;\mathrm{aa}\right\}\right\}$$;

2) Recessive effect:

$$\left\{S_{1}^{j}=\left\{i|X_{i,j}=\mathrm{aa}\right\},S_{2}^{j}=\left\{i|X_{i,j}=\mathrm{AA}\;\mathrm{or}\;\mathrm{Aa}\right\}\right\}$$;

3) Heterozygote effect:

$$\left\{S_{1}^{j}=\left\{i|X_{i,j}=\mathrm{Aa}\right\},S_{2}^{j}=\left\{i|X_{i,j}=\mathrm{AA}\;\mathrm{or}\;\mathrm{aa}\right\}\right\}$$.

For each partitioning strategy, we calculate Δ_WNA_ and its corresponding *p*-value. We repeat the partitioning process for all SNPs, and choose the most significant group partitioning for step one, denoted it as $\left\{S_{1}^{\left(1\right)},S_{2}^{\left(1\right)}\right\}$. In step two, a second SNP *j*′ is considered to further partition the existing two groups into four G-G groups, denoted as $\left\{S_{1}^{\left(2\right)}=S_{1}^{\left(1\right)}\cap S_{1}^{j^{\prime }},S_{2}^{\left(2\right)}=S_{1}^{\left(1\right)}\cap S_{2}^{j^{\prime }},S_{3}^{\left(2\right)}=S_{2}^{\left(1\right)}\cap S_{1}^{j^{\prime }},S_{4}^{\left(2\right)}=S_{2}^{\left(1\right)}\cap S_{2}^{j^{\prime }}\right\}$. Again, the group partitioning that most significantly related to the age-at-disease-onset is selected in the step two. In a similar fashion, the disease-susceptibility SNPs can be selected forwardly into the model to partition samples into different G-G groups. Ten-fold cross-validation (CV) is then used to determine the most parsimonious model with an optimal number of G-G groups. In this procedure, all the subjects are randomly divided into 10 subsets. Then 9 of the 10 subsets are used as the training set, while the remaining one is used as the testing set. The process is repeated 10 times to make sure all subsets have served as a testing set. In each testing set, a test statistic is calculated based on the G-G groups selected from the corresponding training set. The final model with an optimal number of G-G groups is the chosen to be the one that attains the highest significance level of the averaged testing statistic from 10 testing sets. After the final model is determined, an overall test statistic based on entire samples, Δ_WNA_, is obtained, and is used to evaluate the association of the selected G-G groups with the age-at- disease-onset. In order to account for the inflated Type I error due to selection of G-G groups, a permutation test is used to assess the significance level. In the permutation process, the outcomes of individuals (i.e. the age-of-onset and censoring status outcomes) in each permutation replicate are simultaneously permuted. The forward selection algorithm is then applied to the permuted data to choose the best G-G group and calculate Δ_WNA_. By repeating the same process on a large number of permutation replicates (e.g. 1,000), the empirical null distribution of Δ_WNA_ is generated and an empirical *p*-value can be obtained. In a replication study where the G-G group is pre-determined, the asymptotic test based on the Chi-square distribution can be used.

### Modification for population-based case-control studies

Most of existing and ongoing genetic association studies adopt case-control design, where controls are not matched to the cases by age. To facilitate the survival analysis of genetic data from case-control studies, we also propose a modified WNA for case-control data. Suppose the study includes *n* subjects with *n*_1_ cases and *n*_0_ controls (*n* = *n*_1_ + *n*_0_). Assuming the disease has a prevalence of ρ in the general population, we modify the hazard function by adjusting the number of subjects at risk, *Y* (*t*):

$$\Delta {\hat{H}}^{*}\left(t\right)=\frac{\mathrm{\Delta N}\left(t\right)}{Y\left(t\right)+\left(n_{1}/\rho -n\right)}=\frac{\mathrm{\Delta N}\left(t\right)}{Y^{*}\left(t\right)}$$

Correspondingly, we modify the group-wise hazard function by adjusting the number of subjects in group *S*_*l*_,

$$\Delta {\hat{H}}_{l}^{*}\left(t\right)=\frac{\Delta N_{l}\left(t\right)}{Y_{l}\left(t\right)+\left(n_{1}/\;\rho -n\right)\times f_{l}}=\frac{\Delta N_{l}\left(t\right)}{Y_{l}^{*}\left(t\right)}$$

where *f*_*l*_ denotes the frequency of genotype *G*_*l*_ among controls, and $\sum_{l=1}^{L}f_{l}=1$. With this adjustment, we expect to retrieve a pseudo-cohort population with a number of unobserved controls, who are expected to be at risk throughout the study.

## Results

### Simulation studies

In the simulation studies, we evaluated the performance of the proposed WNA approach and compared it with those of Cox-regression-based approaches. Two series of simulations were conducted for perspective cohort studies and case-control studies, respectively. In the simulations, we assumed a subject’s age-at-survey, $T_{i}^{S}$, followed a normal distribution, *N*(60,10^2^), and its age-at-disease-onset, $T_{i}^{O}$, might follow various disease models described below. Each subject had an observed age $T_{i}=\min\left(T_{i}^{O},T_{i}^{S}\right)$, and a censoring status, δ_*i*_, determined by $\delta_{i}=I\left(T_{i}^{O}\leq T_{i}^{S}\right)$. Two causal SNPs with an interaction effect were simulated for each disease scenario. We also assumed each SNP had two alleles, A and a, and the minor allele a had a frequency of 0.3 leading to an early onset of the disease. In addition, eight noise SNPs were also simulated with minor allele frequencies sampled from a uniform distribution, *Unif*[0.1, 0.5]. For each disease model, we simulated one million subjects as the source population, and the disease prevalence was calculated by $\rho =\sum_{i}\delta_{i}/10^{6}$. The parameters were chosen to ensure the disease prevalence was within the range of [0.15, 0.25] in the source population. For prospective cohort studies, 1,000 subjects were selected as the study population, while 500 cases (δ_*i*_ = 1) and 500 controls (δ_*i*_ = 0) were selected for case-control studies.

#### Disease model 1

The underlying disease model was simulated to mimic an ideal scenario where the proportional hazard (PH) assumption was satisfied and the hazard ratio was linear. In the model, we assumed the hazard function *h*_*i*_(*t*) for an individual *i* had a semi-parametric form,

$$h_{i}\left(t\right)=h_{0}\left(t\right)\exp\left(\beta_{1}x_{i1}+\beta_{2}x_{i2}+\beta_{12}x_{i1}x_{i2}\right),$$

where

$$x_{i1}=\left\{\begin{array}{c} 0\begin{array}{ccc} & & \mathrm{if}X_{i1}=\mathrm{AA} \end{array}\\ 1\begin{array}{ccc} & & \mathrm{if}X_{i1}=\mathrm{Aa} \end{array}\\ 2\begin{array}{ccc} & & \mathrm{if}X_{i1}=\mathrm{aa} \end{array} \end{array}\right)$$

and

$$x_{i2}=\left\{\begin{array}{c} 0\begin{array}{ccc} & & \mathrm{if}X_{i2}=\mathrm{AA} \end{array}\\ 1\begin{array}{ccc} & & \mathrm{if}X_{i2}=\mathrm{Aa} \end{array}\\ 2\begin{array}{ccc} & & \mathrm{if}X_{i2}=\mathrm{aa} \end{array} \end{array}\right)$$

In the simulation, we specified the baseline hazard *h*_0_(*t*) by a Weibull distribution with a shape parameter θ = 3 and a scale parameter λ = 100.

#### Disease model 2

The underlying disease model was simulated to mimic a scenario where the PH assumption was met, but the hazard ratio was non-linear. For this model, we assumed the hazard function for an individual *i* had the form:

$$h_{i}\left(t\right)=h_{0}\left(t\right)\exp\left(\beta \cdot I\left(x_{i1}>0\right)\cdot I\left(x_{i2}>0\right)\right).$$

In such a model, we assumed individuals with risk alleles at both loci had a high risk of disease than the remaining individuals.

#### Disease model 3

This disease model mimicked a scenario where the PH assumption was violated. In this model, we assumed the age-at-disease-onset for a subject with a G-G combination of (*x*_*i*1_, *x*_*i*2_) followed a Weibull distribution *W*(λ,θ_*x*i1_, _*x*i12_), where the scale parameter λ was fixed at 100 and the shape parameter varied by the G-G combinations of two causal SNPs. In such a model, we assumed the hazard functions increased over time for all G-G combinations (i.e.,θ > 1).

#### Disease model 4

This disease model also assumed the PH assumption was violated. Different from Disease Model 3, we assumed the hazard functions may remained constant or decreased overtime for certain G-G combinations (i.e. θ ≤ 1), mimicking an early onset disease scenario.

### Simulation results

Simulations were conducted to compare WNA with the conventional Cox regression (COX) approach under the perspective cohort studies. Additional simulations were also perform to evaluate WNA with the modified case cohort (MCC) approach proposed by Nan *et al* (Nan and Lin, 2008) under case-control studies. To be consistent with WNA, we also adopted a forward selection strategy for the COX and MCC, and used the Akaike information criterion (AIC) as the criteria for model selection. For each disease model, we started the analysis with two causal SNPs, and gradually added noise SNPs into the analysis. The simulation was repeated for 1,000 times for each disease model. In each replicate, a final model was selected by each approach, and was then evaluated on an independent sub-cohort of 1,000 subjects from the source population. Because an independent dataset was used for validation in each replicate, the asymptotic test based on the Chi-square distribution was used. The Type I error and power were thus calculated as the probability for the selected final model to have a *p*-value less than 0.05 on the independent sub-cohort of 1,000 subjects.

We first evaluated the Type I errors for both approaches. In this case, we evaluated a null model with ten SNPs, simulated independently from the age-at-disease-onset outcome. As shown in Table 1, the Type I errors were well controlled for COX/MCC approach, and reasonably controlled for WNA. We then evaluated the power and the sensitivity (specificity) of both approaches. The sensitivity was calculated as the probability of selecting a causal SNP in the final model, while the specificity was calculated as the probability of not selecting a noise SNP in the final model. The results for perspective cohort studies were summarized in Table 2. Under the Disease Model 1, while COX had a higher power than WNA (i.e., 0.702 vs. 0.738) when only causal SNPs were considered, its power decreased much faster than that of WNA as the number of noise SNPs increased. When the noise SNPs reaches 8, WNA attained a higher power than COX (i.e., 0.567 vs. 0.427). When the hazard ratio was non-linear (Disease Model 2) or PH assumption was violated (Disease Models 3 and 4), WNA had a consistently higher power than COX. WNA showed the most advantages over COX under Disease Model 4 when the hazard functions do not follow monotonic patterns. In all scenarios, COX tended to have a higher sensitivity, but a lower specificity than WNA. This indicated COX tended to include more noise SNPs in the model than WNA, which partially explained its relatively lower power with the increasing number of noise SNPs. Compared to COX, WNA had relatively more robust performance, especially when the PH assumption was violated.

**Table 1:** Type I errors of WNA and COX/MCC

	**2 SNP**		**4 SNPs**		**7 SNPs**		**10 SNPs**	
	**WNA**	**COX/MCC**	**WNA**	**COX/MCC**	**WNA**	**COX/MCC**	**WNA**	**COX/MCC**
Prospective Cohort Study	0.065	0.045	0.058	0.053	0.059	0.052	0.064	0.047
Case-Control Study	0.032	0.056	0.056	0.053	0.062	0.045	0.058	0.050

**Table 2:** Comparison of WNA and COX in prospective cohort studies

**Disease models**							**Two Causal SNP**		**+ 2 Noise SNPs**		**+5 Noise SNPs**		**+ 8 Noise SNPs**	
							**WNA**	**COX**	**WNA**	**COX**	**WNA**	**COX**	**WNA**	**COX**
Model 1: PH; Linear	θ = 3													
	β_1_ = 0.15					Power:	0.702	0.738	0.641	0.660	0.596	0.532	0.567	0.427
	β_2_ = 0.15					Sensitivity:	0.736	0.924	0.686	0.926	0.633	0.906	0.587	0.906
	β_12_ = 0.15					Specificity	--	--	0.967	0.966	0.942	0.904	0.932	0.847
Model 2: PH; non-linear						Power:	0.802	0.623	0.731	0.513	0.687	0.386	0.661	0.297
	θ = 3					Sensitivity:	0.781	0.818	0.730	0.810	0.681	0.810	0.647	0.810
	β = 0.6					Specificity	--	--	0.975	0.970	0.956	0.905	0.940	0.869
Model 3: Non-PH	θ		AA	Aa	aa									
		BB	3	2.5	2.5	Power:	0.932	0.733	0.910	0.652	0.903	0.522	0.891	0.428
		Bb	3	2	2	Sensitivity:	0.611	0.778	0.588	0.786	0.573	0.786	0.556	0.786
		bb	3	2	2	Specificity	--	--	0.987	0.963	0.974	0.903	0.965	0.843
Model 4: Non-PH	θ		AA	Aa	aa									
		BB	3	3	1	Power:	0.989	0.508	0.979	0.420	0.971	0.304	0.958	0.232
		Bb	3	3	1	Sensitivity:	0.916	0.769	0.851	0.773	0.779	0.773	0.737	0.769
		bb	1	1	0.5	Specificity	--	--	0.897	0.860	0.836	0.799	0.802	0.739

The simulation results for case-control studies were summarized in Table 3. WNA attained a higher power than MCC under all simulated models, which could be explained by the biased estimation of MCC under common disease scenarios. The power of MCC also decreased more rapidly as the number of noise SNPs increased, compared to the power of COX in cohort studies. Similar with COX, MCC tended to have a higher sensitivity, but a lower specificity than WNA.

**Table 3:** Comparison of WNA and MCC in case-control studies

**Disease Models**							**Two Causal SNP**		**+ 2 Noise SNPs**		**+5 Noise SNPs**		**+ 8 Noise SNPs**	
							**WNA**	**MCC**	**WNA**	**MCC**	**WNA**	**MCC**	**WNA**	**MCC**
Model 1: PH; Linear	θ = 3													
	β_1_ = 0.15					Power:	0.762	0.614	0.718	0.434	0.692	0.222	0.674	0.100
	β_2_ = 0.15					Sensitivity:	0.753	0.980	0.717	0.980	0.693	0.978	0.680	0.978
	β_12_ = 0.15					Specificity	--	--	0.926	0.913	0.827	0.763	0.753	0.627
Model 2: PH; non-linear	θ = 3					Power:	0.876	0.561	0.823	0.376	0.838	0.195	0.821	0.076
						Sensitivity:	0.916	0.934	0.909	0.902	0.873	0.902	0.844	0.902
	β = 0.6					Specificity	--	--	0.879	0.878	0.736	0.735	0.605	0.578
Model 3: Non-PH	θ		AA	Aa	aa									
		BB	3	2.5	2.5	Power:	0.926	0.678	0.933	0.480	0.930	0.253	0.932	0.114
		Bb	3	2	2	Sensitivity:	0.753	0.891	0.717	0.891	0.693	0.891	0.680	0.891
		bb	3	2	2	Specificity	--	--	0.926	0.915	0.827	0.777	0.753	0.629
Model 4: Non-PH	θ		AA	Aa	aa									
		BB	3	3	1	Power:	0.987	0.458	0.974	0.277	0.960	0.133	0.952	0.057
		Bb	3	3	1	Sensitivity:	0.971	0.831	0.905	0.831	0.831	0.831	0.769	0.830
		bb	1	1	0.5	Specificity	--	--	0.878	0.917	0.781	0.790	0.729	0.656

For case-control studies, we assumed that the disease prevalence was accurately estimated. However, in practical, the prevalence of a disease might be estimated inaccurately. Therefore, additional simulation was conducted to evaluate the performance of WNA when disease prevalence was estimated inaccurately. For simplicity, we used the Disease Model 2 as an example, and considered both accurate and inaccurately estimated disease prevalence values, including ρ, ρ ± 5%, ρ ± 10%. The results were summarized in Table 4. The power of WNA increased slightly as the disease prevalence decreased. However, the type I errors also increased as the disease prevalence decreased, and would be inflated when disease prevalence was under-estimated. Further, the under-estimation of disease prevalence appeared to increase the sensitivity of SNP selection, but reduced the specificity. By adjusting the disease prevalence, we expect to retrieve a pseudo-cohort population with a number of unobserved controls, who are expected to be at risk throughout the study. Under-estimating the disease prevalence would artificially increase the number of controls in the study, and thus cause bias in the follow-up studies with an independent sub-cohort data from the source population.

**Table 4:** Performance of WNA when disease prevalence is miss-specified

	**ρ-10%**	**ρ-5%**	**ρ**	**ρ + 5%**	**ρ + 10%**
Power	0.858	0.853	0.821	0.784	0.782
Type I	0.108	0.070	0.058	0.051	0.048
Sensitivity	0.912	0.872	0.844	0.818	0.817
Specificity	0.526	0.578	0.605	0.739	0.873

### Application to study the progression to nicotine dependence (ND)

Previous studies have indicated that the progression to ND could be influenced by the interplay of genetic variants [30,31]. Detecting G-G interactions contributing to the development of ND would help to understand the transition process from first cigarette use to nicotine dependence, and to promote the development of early prevention and intervention strategies. For such a purpose, we initiated an interaction search among known ND-associated genetic variants by applying the WNA approach to the Study of Addiction: Genetics and Environment (SAGE) GWAS dataset. The participants of the SAGE were unrelated individuals selected from three large, complementary case-control studies: the Family Study of Cocaine Dependence (FSCD), the Collaborative Study on the Genetics of Alcoholism (COGA), and the Collaborative Genetic Study of Nicotine Dependence (COGEND) [32]. The SAGE included standardized diagnostic assessments of ND by Diagnostic and Statistical Manual of Mental Disorders (DSM) IV, and its assessment plans for age-at-onset variables were also guided by standardized interview protocols and assessments, as described in prior SAGE publications [33,34]. We considered two age-at-onset variables, age-at-onset of ND and age-at-initiation of tobacco uses, and defined progression to ND as their difference. The study subjects under the investigation were limited to those who ever smoked cigarettes daily for a month or more. For non-ND subjects, age-at-survey was used as right-censoring values for age-at-onset of ND. After removing the subjects with missing outcomes, there were 706, 727, and 1,232 subjects in FSCD, COGA and COGEND, respectively. From the literature, we selected 150 SNPs across 64 candidate genes that have been reported for potential association with ND. Among those 150 SNPs, genotypes for 124 SNPs were available in the SAGE dataset, while genotypes for the remaining 26 SNPs were imputed by using PLINK [35]. The HapMap phase III founders of the CEU and ASW populations were used in the imputation as the reference panels for the white and black subjects [36].

**Figure 1:**
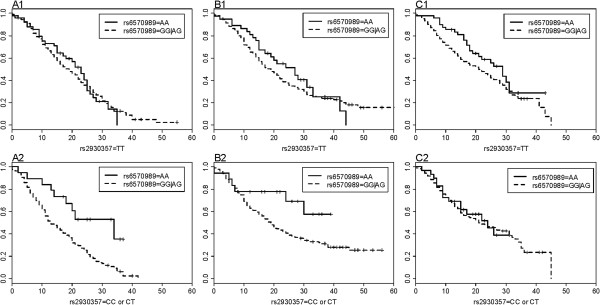
**Survival curves for subjects with different G-G combinations in three studies. A1-A2**. Survival curves for G-G groups in the FSCD study. **B1-B2**. Survival curves for G-G groups in the COGA study. **C1-C2**. Survival curves for G-G groups in the COGEND study.

We applied WNA to FSCD for an initial G-G interaction search and then replicated the initial findings in COGA and COGEND. While applying WNA, we fixed the disease prevalence at ρ = 0.24, which was estimated according to national survey [37]. Two SNPs, rs6570989 (A/G) and rs2930357 (C/T), located in gene *GRIK*2 and *CSMD*1, were identified in the initial search to be jointly associated with progression of ND with a nominal *p*-value of 9.68e-13. Permutation test was then conducted to estimate the empirical *p*-value, accounting for overestimation due to the model selection. The empirical *p*-value obtained from permutation test indicated a significant association (i.e., *p*-value < 0.001). Further validation of the finding in COGA (*p*-value = 0.034) and COGEND (*p-*value = 7.85e-04) showed the association remained significant at 5% level (Table 5). Survival curves in Figure 1 showed that the effect of rs6570989 was modified by the genotypes of rs2930357 in FSCD, which indicated a possible G-G interaction between two SNPs (Figure 1 A1-A2). Similar patterns were also observed in COGA (Figure 1 B1-B2) and COGEND (Figure 1 C1-C2). To account for the possible bias estimation of disease prevalence, we further examined the joint association for the identified two SNPs with the disease prevalence rates of 0.19 and 0.29 (i.e. 0.24 ± 0.05). The results showed that the significance level decreased as the disease prevalence increased (Table 6), but all joint association remained at least marginally significant with the disease prevalence of 0.29 (*p*-values were 4.08e-12, 0.054 and 2.00e-03 in FSCA, COGA and COGEND, respectively).

**Table 5:** Summary of two SNPs identified in FSCD and replicated in COGA and COGEND

**SNP**	**Allele**	**Chro**	**Position**	**Gene**	**Grouping**	***p-*** **values**
rs6570989	A/G	6	101957413	*GRIK*2	{AA}{AG,GG}	FSCD: 9.68e-13
rs2930357	C/T	8	3709660	*CSMD*1	{TT}{CC,CT}	COGA: 0.034
						COGEND: 7.85e-04

**Table 6:** Evaluating the joint association of two SNPs with varied disease prevalence rates

**Disease Prevalence**	**ρ = 19%**	**ρ = 29%**
*P-*values	FSCD: 2.41e-13	FSCD: 4.08e-12
	COGA: 0.021	COGA: 0.054
	COGEND: 2.97e-04	COGEND: 2.00e-03

We also applied the Cox regression method to the same datasets. Only pair-wise interactions among SNPs were considered in the selection. The final model was determined by forward selection to minimize the AIC value. In FSCD, Cox regression with forward selection picked up nine SNPs involving a complicated model with a total number of 45 parameters. This association could not be replicated in either COGA (*p*-value = 0.703) or COGEND (*p-*value = 0.218).

## Discussion

Complex diseases, manifesting with various clinical features, are believed to be caused by the joint action of multiple genetic variants through distinctive biological pathways. If two genes are jointly involved in producing the variability of a disease feature, whether additively or not, biological interaction between them is involved [38]. Although there is growing interest in detecting genetic variants that characterize disease progression, relatively few approaches have been proposed to evaluate the interaction among multiple genetic variants. In this article, we have proposed a non-parametric approach, referred to as WNA, for testing the joint association of multiple genetic variants with the age-at- onset outcomes, taking possible G-G interactions into account. The approach can be applied to both prospective cohort studies and case-control studies. Through simulations and an empirical study, we have shown that our approach had a comparable or better performance than Cox regression under various scenarios. The outperformance of WNA over Cox regression can be explained by the following reasons: 1) WNA does not assume any patterns of the hazard functions among G-G groups, which makes it more robust under various disease scenarios. While we expect Cox regression to have a better performance than WNA when the underlying disease model is known, in reality our understanding of the mode of inheritance for complex diseases is very limited. In such a case, non-parametric approaches, such as WNA, would have more advantages for the search of gene-gene interactions. 2) When a set of SNPs are involved, Cox regression tends to select highly complex genetic models with a large number of parameters. Unlike Cox regression, WNA is a non-parametric approach, and does not assume any parametric model for the selected G-G combinations. When the assumptions are violated, WNA likely captures the underlying G-G combinations, which can be replicated in independent studies. 3) Given the disease prevalence in the source population, WNA can be easily extended to studies with case-control designs. For case-controls studies, it has been suggested that Cox regression should be used with great caution [17,18], due to the biased estimation of the effect size and incorrect statistical inference.

In the simulation, one of our aims is to examine robustness of the proposed WNA approach under case-control studies of common diseases, where the COX/MCC approach has biased estimates. Therefore, we have evaluated the performance of two approaches with an independent sub-cohort dataset from the source population, mimicking an independent follow-up study in a real application. The results have shown WNA approach had an improved power by adjusting for the disease prevalence in case-control studies, but in the meantime, under-estimating disease prevalence may lead to an inflated type I error for WNA. Caution should be taken in specifying the disease prevalence in real applications.

Another important consideration is the choice of weight function in WNA. The weight function in this study gives the most weight to departures of hazard functions at early ages. Alternatively, we could also adopt a general class of weight functions based on the Kaplan-Meier estimator [28]. The weight function has the form,

$$W_{p,q}\left(t\right)=\hat{S}{\left(t-\right)}^{p}{\left[1-\hat{S}\left(t-\right)\right]}^{q},p\geq 0,q\geq 0,$$

where $\hat{S}\left(.\right)$ is the Kaplan-Meier estimator of the survival function. The values of *p* and *q* should be chosen appropriately according to the hypothesis of interest. For instance, when *p* = 0, *q* > 0, the above weight gives more consideration to departures of hazard functions at late ages.

In the real data application, we identified two SNPs, rs6570989 and rs2930357, jointly associated with ND. In a recent GWAS of 3497 Dutch subjects, both of these two SNP were found to be significantly associated with current smoking [39]. These two SNPs are located in gene *GRIK*2 and *CSMD*1, respectively. Both *GRIK*2 and *CSMD*1 have been suggested to be functionally related with ND. *GRIK*2 belongs to the kainate family of glutamate receptors, which are actively involved in a variety of neurophysiologic processes [40,41]. *GRIK*2 has also been reported to be associated with smoking cessation [42]. Gene *CSMD*1 was shown to be highly expressed in the central nerve system [43], and to be related to smoking cessation [44]. A number of studies have also suggested that early smoking initiation and the development of nicotine dependence are associated with greater difficulty to quit smoking [45-47]. Nonetheless, relatively few studies have been conducted to evaluate ND age-at- onset outcomes, and our knowledge regarding the genetic contribution to the progression of ND is still lacking. While it is biologically plausible that the two identified genes may have a joint association contributed to the progression of ND, further studies are required to replicate this result.

We are aware that the proposed approach has some limitations. First, the test statistic of WNA follows an asymptotic Chi-square distribution when evaluating common genetic variants. However, if a genetic variant has a very low minor allele frequency, it may form certain G-G groups with a small number of subjects. In such a case, the asymptotic property of the test may not hold [48,49]. Therefore, for the rare variants, we suggest that an exact test be used to evaluate the significance [50]. Second, the proposed approach used a forward selection strategy, and we expect the power to decrease if none of the genetic variants has any marginal effect. In this specific case, exhaustive selection will be needed to detect a G-G interaction, but at a much higher computation cost. Third, the proposed approach is implemented in R with model selection, cross-validation and permutation procedures. It is less computationally efficient than applying a Cox regression model available in R. On a dual core 3.20GHz desktop, the average computation time for applying the WNA approach and Cox regression were 23.2 second and 0.26 second, respectively. On replication datasets when the optimal G-G model was pre-determined, the computation time for applying the WNA approach was significantly reduced to 0.028 second, which was comparable to those for Cox regression (0.031 second).

One major advantage of the proposed WNA approach is its capability of handling multiple genetic variants with the consideration of possible high-order G-G interactions [51]. It is worthwhile to note that WNA is a nonparametric approach developed for both cohort studies and case-control studies, which differs from other approaches, such as the kernel-machine based approach [52]. Further, we limited the application of WNA approach to population-based case-control studies in which the cases and controls were not matched by age. If controls are matched to cases and are randomly selected from all those at risk at the age-of-onset of the cases, Cox regression can estimate the effect size of by a conditional likelihood method without bias [53,54].

## Conclusions

We have proposed a statistical approach for detecting genetic interactions associated with age-at-onset outcomes. The approach is able to capture high-order gene-gene interactions, and can be applied to both prospective cohort studies and case-control studies. Through simulations, we showed that the new approach had comparable or better performance than the conventional Cox-regression-based methods. The empirical data applications to nicotine dependence also identified two genes, *GRIK2* and *CSMD1*, joint associated with the progression of nicotine dependence. In addition to conventional statistical approaches for survival outcomes, the new approach provides an alternative way to model genetic interactions related to survival outcomes.

## Competing interests

The authors declare that they have no competing interests.

## Authors’ contributions

ML designed the study, performed the analysis and wrote the manuscript; JCG participated in methodology development and wrote the manuscript; NB participated in data interpretation and wrote the manuscript; JCA participated in data interpretation and wrote the manuscript; QL conceived the idea, designed the study and wrote the manuscript. All authors read and approved the final manuscript.
